# Gender-Dependent Modulation of Alzheimer’s Disease by Brain Ischemia. Comment on Lohkamp et al. Sex-Specific Adaptations in Alzheimer’s Disease and Ischemic Stroke: A Longitudinal Study in Male and Female APP_swe_/PS1_dE9_ Mice. *Life* 2025, *15*, 333

**DOI:** 10.3390/life15071146

**Published:** 2025-07-21

**Authors:** Ryszard Pluta

**Affiliations:** Department of Pathophysiology, Medical University of Lublin, 20-090 Lublin, Poland; pluta2018@wp.pl

**Keywords:** Alzheimer’s disease, brain ischemia, gender, female, male, mechanisms

## Abstract

This comment focuses on the contribution of experimental brain ischemia to the overwhelming incidence of Alzheimer’s disease in women as presented by Lohkamp et al. in *Life* 2025, 15, 333. The authors showed that in Alzheimer’s disease and ischemic stroke there are sex-dependent adaptations in the form of cross-links and vice versa. It was emphasized that the high longevity of women in itself does not explain the mechanisms underlying the biological differences between the sexes causing a female predominance in the development of Alzheimer’s disease. Differences were demonstrated between males and females: female APP/PS1 mice had greater amyloid deposition, hyperactivity, lower body weight, and reduced cerebral blood flow, as well as less neuroinflammation, which the authors suggest may have potential neuroprotection. It should be noted that some of the information presented in the article by Lohkamp et al. raises more questions than answers. Therefore, future studies should consider, for example, studies using single-cell technologies that can provide insight into the timing and sequence of cellular dysfunctions across sexes and analyze the continuity of changes over time, starting from short-term observations of a few days and ending with long-term observations of a year or more, to assess the continuity and differentiation of changes.

In recent years, aging societies have shown an increasingly strong link between Alzheimer’s disease and the cumulative effects of brain ischemia [1,2,3,4]. Brain ischemia and Alzheimer’s disease pose a huge challenge to global healthcare. The lifetime risk of developing any of these diseases is 50% for women and 30% for men [5]. The neuropathology of Alzheimer’s disease and post-ischemic brain is characterized by the growth of amyloid plaques and neurofibrillary tangles, leading to brain atrophy caused by the progressive loss of neuronal cells, synapses, and ultimately the gradual development of dementia [6,7,8,9,10,11]. Estimates suggest that a new diagnosis of dementia is made every three seconds worldwide, with Alzheimer’s disease accounting for more than half of these cases [12]. Both women and men are born without senile plaques and neurofibrillary tangles, but advanced Alzheimer’s disease is characterized by the presence of both types of changes and affects mainly women. Autopsy studies of the brain have shown that 80% of patients with Alzheimer’s disease have small vessel damage, and 42–50% of them have brain ischemia or brain microinfarctions, suggesting that cerebral vascular dysfunction is an important component of this disease [13,14,15]. Brain ischemia doubles the risk of Alzheimer’s disease and vice versa [16,17]. Cerebrovascular dysfunction resulting in ischemic episodes is now widely recognized as an etiological factor of Alzheimer’s disease, driving the pathogenesis and progression of amyloid and tau protein [1,2,3,4]. It is known that in the aging brain, many imperceptible, minor ischemic events occur over the years and become more noticeable over time [18,19]. These small ischemic strokes have the same cause as large, symptomatic strokes, but they affect much smaller vessels and the volume of brain parenchyma surrounding them. However, this injury likely accumulates over time [18,19], contributing to the onset and progress of Alzheimer’s disease [1,2,3,4]. In this context, it has been presented that brain ischemia take part in driving tau protein and amyloid during neurodegeneration in Alzheimer’s disease [4,6,7,8,9]. Clinical observations have shown that Alzheimer’s disease is a risk factor for brain ischemia and vice versa, which suggests that identical or strictly associated processes may contribute to the slow progression of both diseases [13]. Experimental investigations have also shown a connection between brain ischemia and Alzheimer’s disease, whereby brain ischemia causes an augmented risk of cognitive impairment and the progress of Alzheimer’s disease dementia [2,20,21,22,23]. The clinical signs vary depending on the extent of the injury, and ranges from acute symptoms immediately after extensive ischemic brain changes to an insidious onset lasting many years in the form of the prodromal form of Alzheimer’s disease pathology [10,13].

Accumulating evidence suggests that gender has a differential effect on the incidence and severity of brain ischemia and Alzheimer’s disease. Men are more susceptible to brain ischemia at a younger age, whereas women are more susceptible after menopause [24]. Because it is well known that brain ischemia is a sexually dimorphic disease, emphasis is placed on the need to better understand the mechanisms underlying differences in the pathophysiology of stroke, as well as how current treatment options affect both sexes, in order to improve the care of women after stroke [25]. Gender differences have been reported in the epidemiology, risk and response to stroke [25]. Moreover, the results of in vitro ischemia experiments have shown that male and female cells do not respond in the same way to signals of death or survival after injury [26].

In the case of Alzheimer’s disease, almost two-thirds of patients are women, not only because of their longer life expectancy but also because of other distinct biological and psychological risk factors that promote disease progression in them [27,28]. Interestingly, the first recorded case of Alzheimer’s disease was a woman. Gender differences in life expectancy therefore contribute to women’s increased risk of dementia, as women worldwide live on average 4–7 years longer than men [29,30]. Despite prolonged survival with Alzheimer’s disease [30,31], women have exhibited an increased global neuropathological burden of Alzheimer’s disease and more severe cognitive impairment [32]. Moreover, women with Alzheimer’s disease show poorer cognitive performance than men, despite similar levels of brain atrophy [33]. As a result, women with Alzheimer’s disease experience more disability-adjusted life years due to the disease than men [30]. The longstanding phenomenon in which women exhibit higher morbidity but lower mortality compared with men as they age, regardless of diagnosis, is known as the “male-female health-survival paradox” [34]. However, there is a lack of data on how sex differences contribute to the differential effects of brain ischemia on cognitive impairment in Alzheimer’s disease, especially in the context of the comorbidity of these diseases.

Current knowledge indicates that post-ischemic neuropathology and Alzheimer’s disease-related neuropathology independently but jointly contribute to cognitive decline and dementia [11,20,21,22,23]. Although it is known that Alzheimer’s disease and brain ischemia coexist and that they potentially contribute to cognitive decline, a full understanding of their pathophysiological overlap remains an enigma. The mechanisms underlying these biological sex differences are still not fully understood, and high life expectancy in women alone does not explain them. Therefore, the influence of brain ischemia on the modulation of Alzheimer’s disease pathology in a sex-dependent manner has recently been intensively studied in order to explain the predominance of this disease in women [2,17].

It was initially hypothesized that brain microinfarcts play a crucial role in the early development of Alzheimer’s disease-like pathology in a sex-dependent manner in young APP/PS1 mice [2]. The results showed that microinfarcts reduced amyloid deposits without affecting soluble amyloid levels in the brain of male and female APP/PS1 mice with 1-month survival, while causing rapid and long-lasting cognitive deficits in males and mild and transient cognitive decline in females. In male APP/PS1 mice, microinfarcts induced acute cerebral hypoperfusion followed by chronic hyperperfusion [2]. In female APP/PS1 mice, microinfarcts also induced hypoperfusion, which resolved in the chronic phase. The authors demonstrated that microinfarcts induced strong microglial activation and recruitment of peripheral monocytes to lesion sites and amyloid plaques in female APP/PS1 mice, which was likely responsible for reduced amyloid deposition [2]. Finally, Dickkopf-1, which plays a key role in synaptic and neuronal dysfunction in Alzheimer’s disease, was strongly induced at lesion sites in male APP/PS1 mice, whereas its induction was reduced in females [2].

In contrast, a study by Lohkamp et al. published in Life [17] on the long-term effect of brain ischemia on the progression of Alzheimer’s disease in APP/PS1 mice showed deterioration of cognitive functions and white matter degeneration. APP/PS1 mice, despite early hypertension, showed sporadic hypoperfusion and thickened cerebral cortex and hippocampal hypertrophy [17]. The authors interpreted this phenomenon as likely being the result of amyloid accumulation and neuroinflammation. Therefore, the first question that arises for the authors is: was the number of neurons and their condition taken into account, which should be understood as hippocampal hypertrophy, or did the authors mean the volume of the hippocampus, which increased for the reasons given above? According to the authors, mice subjected to brain ischemia preserved cognitive abilities despite thinning of the cerebral cortex and atrophy of the hippocampus. The authors attribute this to cerebral vascular adaptations, including increased blood flow in the hippocampus and thalamus [17]. If hippocampal atrophy occurs, there are no neurons in it or only remnants of them. What happened to the accumulated amyloid? Is it possible that amyloid could be cleared from the hippocampus by increased activity of the low-density lipoprotein-receptor-related protein 1 gene caused by ischemia [35]? Regarding neuronal loss, perhaps in this situation we should take into account neurogenesis, which we observed after brain ischemia in a rat with a 1-year survival [36]. The authors concluded that ischemia does not worsen the course of Alzheimer’s disease. Further, how does it relate to existing data; also, the authors mention in their work that they influence each other [2,16,17]. The authors found sex differences: female APP/PS1 mice had “more severe amyloid deposition, hyperactivity, lower body weight, and reduced cerebral blood flow but less neuroinflammation, suggesting potential neuroprotection”. What does the term “more severe amyloid deposition” mean? “Reduced cerebral blood flow and less neuroinflammation, suggesting potential neuroprotection” in what mechanism? Is it possible this is by cleansing amyloid from the brain via increased activity of the low-density lipoprotein-receptor-related protein 1 in the hippocampus, as has been shown after brain ischemia with long-term survival [35]? The authors conclude that their results indicate that white matter degeneration and amyloid pathology are major contributors to cognitive decline in Alzheimer’s disease, with ischemia-related deficits being attenuated by cerebrovascular adaptation [17]. What does the above statement mean and by what mechanism does this occur?

Housing animals individually in cages has a significant impact on their behavior. It is recommended that at least two animals be housed in each cage. The authors stated that the experiments were conducted using a double-blind design. This should have been clearly explained as to who knew what was being done and who did not. Furthermore, the authors disclosed that data loss occurred, which is possible, but is another drawback of the presented study. The work contains many speculative statements, so drawing any far-reaching conclusions is simply excessive.

The authors may rely on indirect evidence, partially consistent with their results, derived from clinical and experimental studies regarding the potential role of polo-like kinase 2 in the regulation of amyloid precursor protein phosphorylation and toxicity. Namely, inhibition of polo-like kinase 2 prevented cognitive decline in a sex-dependent manner in male APP/PS1 mice [37]. Surprisingly, cellular analysis showed that treatment with a polo-kinase 2 inhibitor increased the burden of amyloid plaques and increased the levels of soluble β-amyloid peptide 1-40 and β-amyloid peptide 1-42 in the cerebral cortex, as well as insoluble β-amyloid peptide 1-42 in the hippocampus of female mice, without affecting the pathology of amyloid precursor protein in males [37]. These results indicate the potential of polo-kinase 2 inhibition to alleviate cognitive symptoms in males [37]. However, paradoxically, it exacerbates amyloid pathology in females by increasing the amyloidogenic processing of amyloid precursor protein, which creates a controversial aspect of its therapeutic effect in females. Overall, these data underline the sex-dependent nature of the effects of polo-like kinase 2 inhibition, which may also be influenced by the genetic background of the transgenic mouse model used [37]. This suggests that tau protein behavior should also be considered in the interpretation of the results, which the authors did not do. Furthermore, the behavior of apolipoprotein E, which is considered a greater risk factor for developing Alzheimer’s disease in women than men should have been assessed. This is explained by the interaction between apolipoprotein E and estrogen in women [38].

The final recommendations of Lohkamp et al. [17] are that gender-specific therapies are crucial for Alzheimer’s disease and brain ischemia. I believe that these are far-reaching recommendations for which there is no hard evidence, and their justification is based on contradictory and controversial results and has no basis to currently revolutionize the changes in treatment that we are really striving for. It is known that there is a certain intermediate phase in which neuropathological changes and their component proteins accumulate, and that there may be a certain mathematical threshold for the female population that is characterized by an excessive tendency to develop the clinical form of Alzheimer’s disease in women. In order for the authors’ research-based suggestions to be translated into clinical practice, they must be confirmed by numerous multi-center clinical trials indicating the potential for transferability of the knowledge to the clinic. I think the authors point to the beginning of this path, which cannot be taken away from them. Although the theoretical foundations and general knowledge about the predominance of Alzheimer’s disease in women seem to be quite solid, their implementation into clinical practice is definitely premature.

Research shows that the goal is defined, set and clear [17], but its achievement is currently associated with many problems and doubts that may be solved in the near future. The proposed work will define the directions of future research, pending the development of modern research techniques that will enable personalized treatment. However, there is still a long way to go. In studies at this stage, the problem is not the small groups, but the lack of certain groups, e.g., those with ovariectomy. Future studies should also examine and take into account blood amyloid, tau protein, inflammatory factors and estrogen levels. When considering the survival time of mice, it is important to consider how genes related to amyloid precursor protein metabolism and, for example, inflammation behave over time in this study, as well as when brain ischemia is added as additional injury. Do not the mice go through menopause at about one year of age? A bias in the study was the exclusion of mice with complete hippocampal atrophy from the analysis, because we know that in humans we observe heterogeneity in Alzheimer’s disease neuropathology. Hippocampal atrophy appears to be related to individual sensitivity. Moreover, there is a surprisingly high mortality rate in the study, which the authors reported in their response to the reviewer and presented in Supplementary Table S1. This issue was not addressed in the article and is not necessarily available to the reader. Furthermore, the authors revealed in their response to the review and Supplementary Table S1 that in the presented mouse studies epileptic seizures were observed, an additional factor complicating the experiments and their interpretation. The use of SEM in data calculations confirms the large discrepancy in results and the above facts; however, this can only be noticed by an experienced researcher. Research in this area is not perfect, and the groups are probably very diverse in terms of health status or additional pathologies, which does not make it easier to interpret the results obtained and draw conclusions, which I understand, of course. Moreover, the information presented in the work and the responses to the reviews raise more questions than answers for now. Finally, it should be stated with full responsibility that the interpretation of the data by the authors is highly controversial and speculative.

Over the past century, technological advances have greatly enhanced our ability to test for and diagnose Alzheimer’s disease, and future technological advances will likely resolve the gender issue in the disease. Imaging techniques such as MRI and PET now allow for early detection of the disease, and research on biomarkers in cerebrospinal fluid and blood has revolutionized the time and accuracy of diagnosis [39]. What once required post-mortem brain analysis can now be identified years before symptoms appear, offering hope for earlier treatment. Despite diagnostic advances, many years of research into the etiology of Alzheimer’s disease have not yielded clear answers. The disease remains incurable, and millions of people around the world struggle with its ongoing progression.

It appears that long-term studies using single-cell technologies may provide deeper insight into the timing and sequence of cellular dysfunction, enabling more precise identification of early biomarkers and potential therapeutic windows. Another very important area of research is understanding the role of the brain microenvironment in Alzheimer’s disease. Although we know that neurons, glial cells, and blood vessels play distinct roles in disease progression, the interactions between these cell types and their modulation by the surrounding microenvironment are also important. Advances in spatial transcriptomics combined with single-cell analysis promise to reveal how the local tissue environment influences cell behavior and disease progression across sexes. It not only explores the role of neurons, glial cells, and blood vessels in Alzheimer’s disease, but also provides key insights into the complex interactions in the brain’s microenvironment—an area that has historically remained underexplored. These approaches may open new avenues for understanding the progression of Alzheimer’s disease and the possible potential for new therapeutic interventions aimed at restoring brain homeostasis, in particular by targeting specific cell types and molecular pathways disrupted in Alzheimer’s disease in relation to gender.

I fully agree with the authors that the future of drug development for Alzheimer’s disease must be based on a multifaceted approach that integrates immunotherapy with new strategies that address other aspects, not only targeting amyloid and tau protein, but also mitochondrial and synaptic dysfunction, neuroinflammation, the brain–gut axis, genetic risk factors, and sex-specific pharmacogenomics. As research progresses, the synergy of these therapeutic approaches could provide the breakthroughs needed to effectively combat Alzheimer’s disease and improve outcomes for patients worldwide [39,40]. In addition, in this experimental system, studies of gene and protein changes in single brain cells, especially depending on gender, should be included. Although technical limitations currently make it difficult to properly interpret the studies presented by Lohkamp et al., [17] this does not indicate their impracticability. Sometimes you can learn more from failures than from successes. In this regard, Lohkamp et al. [17] showed that more rigorous studies are needed to definitively demonstrate whether cerebral ischemia can effectively modify the course of Alzheimer’s disease with respect to gender. Unfortunately, it must be said that their current research raises more questions than it answers. However, we can draw many future lessons from this preclinical failure. The authors’ efforts can be summarized as follows: As we move forward, the achievements of the early pioneers in Alzheimer’s disease research serve as a reminder that scientific discovery depends on the persistence of those who courageously ask questions, observe, and innovate. Further work using the modern techniques presented above may help clarify this research issue. Although I have my doubts about the authors’ suggestions, I believe that the work was not wasted, and I know that taking the first steps is always difficult. Given the growing interest in the presented issues and the emergence of many modern techniques, this opens up enormous opportunities for future research. Regardless, I appreciate the enormous amount of work that went into the study and congratulate the authors for having the courage to take on such a difficult, complicated and controversial topic.
